# Association between the triglyceride-high-density lipoprotein-glucose-body index and atrial fibrillation: a retrospective case-control study

**DOI:** 10.3389/fnut.2026.1940518

**Published:** 2026-09-23

**Authors:** Yunling Geng, Xuebo Li, Yang Yang, Chaoqun Huang, Shangzhi Shu, Shuyan Li

**Affiliations:** Department of Cardiovascular Disease, The First Hospital of Jilin University, Changchun, China

**Keywords:** atrial fibrillation, insulin resistance, obesity, TyG, TyHGB

## Abstract

**Background:**

The triglyceride-glucose (TyG) index is a surrogate marker of insulin resistance and has been associated with atrial fibrillation (AF) risk, but it does not capture adiposity. The triglyceride-high-density lipoprotein-glucose-body (TyHGB) index, which integrates lipid profile, glucose metabolism, and body mass index, may provide a more comprehensive metabolic assessment. However, evidence regarding its association with AF remains limited.

**Methods:**

This retrospective case-control study included 1,137 participants (314 non-AF controls, 414 paroxysmal AF, and 409 persistent AF). Multivariable logistic regression was performed using three hierarchical models. Restricted cubic splines were used to examine non-linear relationships. Receiver operating characteristic curves were generated to compare the predictive performance of TyHGB, TyG, and their combination.

**Results:**

Both TyHGB and TyG were independently associated with AF risk across all models (all *P* < 0.001). In the fully adjusted model, each one-unit increase in TyHGB was associated with a 61% increase in the odds of AF (OR 1.61, 95% CI 1.41–1.85). Significant non-linear dose-response relationships were observed for both indices (*P* for non-linear < 0.001). The predictive performance of TyHGB was comparable to TyG (AUC 0.716 vs. 0.717, *P* = 0.902). The combined model achieved the highest AUC (0.723) and significantly outperformed TyG alone (*P* = 0.002), though it was not superior to TyHGB alone (*P* = 0.366).

**Conclusions:**

TyHGB is a novel, simple, and low-cost metabolic index independently associated with AF risk, with predictive performance comparable to TyG. It may serve as a convenient complementary marker for metabolic risk assessment in clinical practice.

## 1 Introduction

Atrial fibrillation (AF) is the most common sustained cardiac arrhythmia and is associated with an increased risk of stroke, heart failure, and mortality worldwide (1, 2). With the aging population and increasing prevalence of obesity, diabetes, and metabolic syndrome, the global burden of AF continues to rise, underscoring the need for effective risk stratification and early identification of high-risk individuals (3, 4). Identifying simple, cost-effective, and readily available biomarkers that capture metabolic dysregulation is therefore a priority for AF prevention. Early detection of metabolic abnormalities could enable timely lifestyle and pharmacological interventions, potentially reducing the incidence of AF and its associated complications (5).

The triglyceride-glucose (TyG) index, calculated from fasting triglycerides and glucose, has emerged as a convenient surrogate marker for insulin resistance and has been associated with AF risk in various populations (6–8). Its simplicity and low cost make it particularly attractive for large-scale screening and resource-limited settings (9). However, TyG does not incorporate adiposity, a well-established independent risk factor for AF that contributes to atrial remodeling through inflammation, oxidative stress, and hemodynamic changes (10–12). Excess body weight, particularly visceral obesity, promotes epicardial adipose tissue (EAT) accumulation, which secretes pro-inflammatory adipokines that can directly infiltrate the atrial myocardium and create a pro-arrhythmogenic substrate (13, 14). EAT volume has been shown to correlate with AF burden and recurrence after ablation (15, 16). Consequently, relying solely on TyG may not fully capture the comprehensive metabolic burden imposed by excess body weight, highlighting the need for a more integrated metabolic index.

The triglyceride-high-density lipoprotein-glucose-body (TyHGB) index is a novel composite metabolic indicator that integrates the triglyceride-to-high-density lipoprotein cholesterol ratio (TG/HDL-C), fasting blood glucose (FBG), and body mass index (BMI). By combining these three dimensions, lipid profile, glucose metabolism, and adiposity, TyHGB may provide a more comprehensive assessment of metabolic dysregulation relevant to AF risk. The TG/HDL-C ratio has been recognized as a reliable marker of insulin resistance, particularly in populations with metabolic syndrome, while BMI is the most widely used anthropometric measure in clinical practice (17). The rationale for incorporating BMI is that adiposity and insulin resistance often coexist and synergistically contribute to atrial remodeling through overlapping inflammatory pathways (18–20). Thus, TyHGB may capture the cumulative and potentially synergistic effects of concurrent metabolic abnormalities, hyperglycemia, dyslipidemia, and obesity, which could have multiplicative rather than additive effects on AF risk. Nevertheless, evidence regarding the association between TyHGB and AF, as well as its comparative performance with the established TyG index, remains limited. To date, no study has systematically evaluated the relationship between TyHGB and AF risk, nor has the dose-response pattern been characterized.

Using a retrospective case-control study, we aimed to: (1) evaluate the association between TyHGB and AF risk; (2) explore the dose-response pattern using restricted cubic splines; and (3) compare the discriminative ability of TyHGB, TyG, and their combined model.

## 2 Methods

### 2.1 Study design and population

In this retrospective analysis, we reviewed the clinical records of all consecutive patients who were admitted to the Department of Cardiovascular Disease at the First Hospital of Jilin University between January 1, 2023 and May 30, 2024, and who had a primary diagnosis of AF or paroxysmal supraventricular tachycardia (PSVT). A total of 1,500 patients met these preliminary screening criteria during the study period. After applying the pre-specified diagnostic criteria and exclusion criteria, 1,137 patients were included in the final analysis as shown in Figure 1. Among them, 409 patients had persistent AF, 414 had paroxysmal AF, and 314 patients with a primary diagnosis of PSVT served as the control group. The control patients were recruited consecutively during the same study period and were frequency-matched to the AF groups by age. Notably, we did not perform strict 1:1 individual matching to preserve sample size and statistical power for subgroup analyses. Instead, frequency matching by age was applied, and multivariable logistic regression was used to adjust for remaining imbalances in baseline characteristics. Control patients were confirmed to be free of AF based on three criteria: no documented history of AF, no evidence of AF on standard 12-lead electrocardiogram, and no evidence of AF on 24 h Holter monitoring. The sample size was therefore determined by the availability of eligible cases during the study timeframe, rather than by a prospective power calculation.

**Figure 1:**
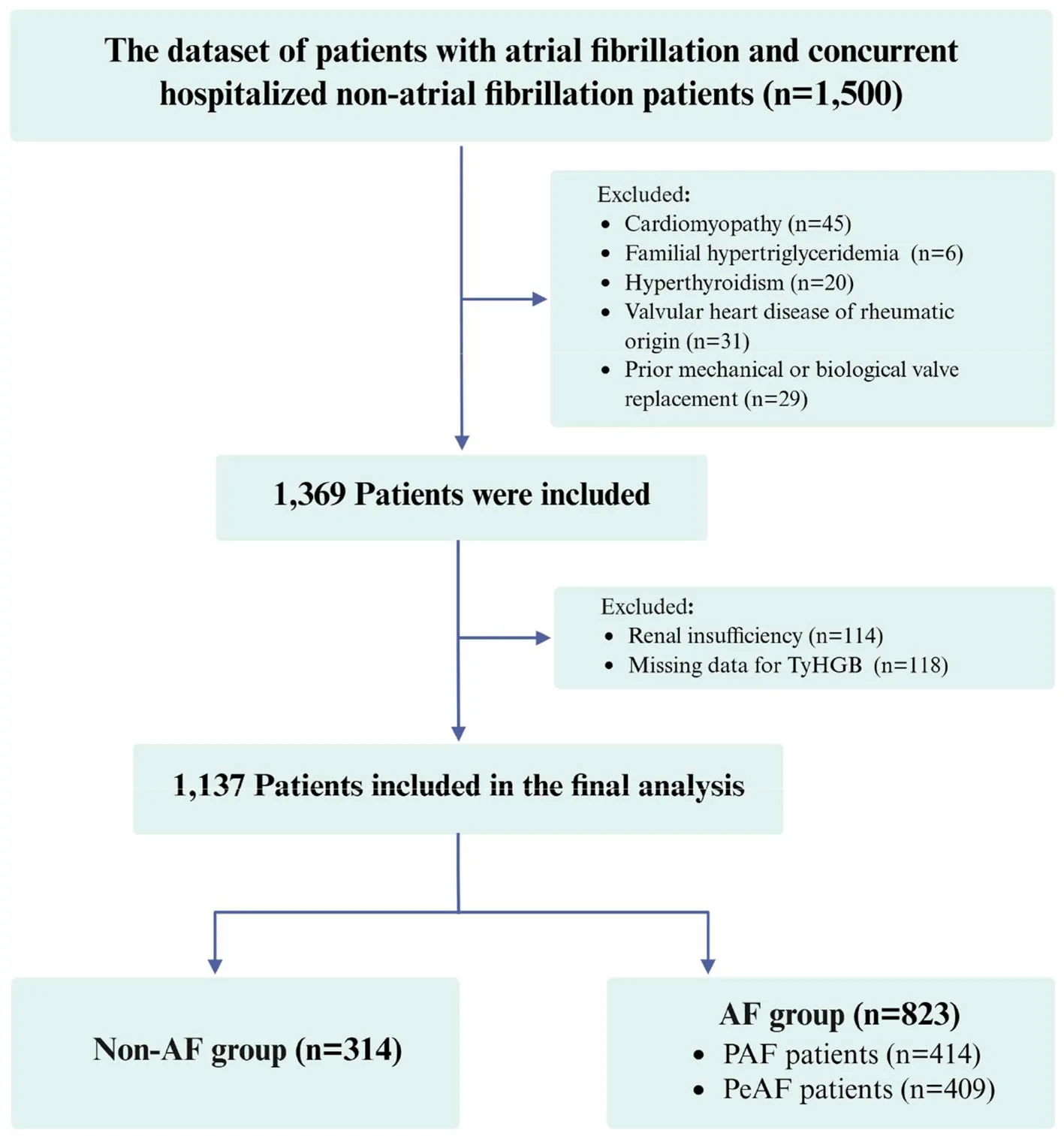
Study population selection flowchart.

The data were accessed for research purposes on May 1, 2026, and all patient information was anonymized. Exclusion criteria included: (1) rheumatic valvular heart disease; (2) cardiomyopathy; (3) congenital heart disease; (4) chronic pulmonary heart disease; (5) prior mechanical or biological valve replacement; (6) hyperthyroidism; (7) familial hypertriglyceridemia. Patients with incomplete TyHGB data or impaired renal function were also excluded. Additionally, only patients who were in a clinically stable condition at the time of admission were considered for enrollment; those with acute coronary syndrome, acute decompensated heart failure, or acute infectious or inflammatory conditions were not included in the initial screening. The study protocol was approved by the Ethics Committee of the First Hospital of Jilin University (No. 2026-381). Written informed consent was waived due to the retrospective design.

### 2.2 Data collection and definitions

Demographic characteristics and clinical parameters were systematically retrieved from inpatient electronic medical records and entered into a pre-designed Excel database. Fasting venous blood samples were routinely collected from all patients after an overnight fast of at least 8 h, on the morning following admission. The collected variables were classified into four domains. Demographics: age, gender, height, weight, smoking, and drinking. Comorbidities: hypertension, diabetes mellitus (DM), coronary heart disease (CHD), and stroke. Laboratory parameters: aspartate aminotransferase (AST), alanine aminotransferase (ALT), total cholesterol (TC), triglycerides (TG), high-density lipoprotein cholesterol (HDL), low-density lipoprotein cholesterol (LDL), serum creatinine (SCr), uric acid (UricAcid), FBG, red blood cell count (RBC), hemoglobin (HGB), mean corpuscular volume (MCV), platelet count (PLT), and mean platelet volume (MPV). Echocardiographic parameters: left ventricular ejection fraction (LVEF), left ventricular end-diastolic dimension (LVEDD), left atrial diameter (LAD), and left ventricular diastolic dysfunction (LVDD).

All patients with suspected or documented AF underwent a standard 12-lead electrocardiogram and 24 h Holter monitoring. AF was diagnosed based on the 2020 ESC Guidelines for the diagnosis and management of AF, defined as an irregularly irregular rhythm with absence of distinct P-waves and an irregular ventricular rate, documented on a standard 12-lead electrocardiogram or on a rhythm strip of at least 30 seconds (2). The diagnosis of AF was confirmed by two independent cardiologists based on all available electrocardiogram and Holter recordings for every patient. AF subtypes were classified according to established clinical guidelines: PAF was defined as AF episodes lasting ≤ 7 days, whereas PeAF was defined as episodes lasting > 7 days (2). Left ventricular diastolic dysfunction (LVDD) was assessed by a trained cardiac ultrasound technician according to the 2016 recommendations of the American Society of Echocardiography and the European Association of Cardiovascular Imaging (21). Control patients also underwent 24 h Holter monitoring as part of their routine clinical evaluation to confirm the absence of AF.

BMI was calculated as weight divided by height squared (kg/m^2^). The TyG index was calculated as: TyG = ln[TG (mg/dL) × FBG (mg/dL) / 2] (22). The TyHGB index was calculated as: TyHGB = TG (mmol/L)/HDL (mmol/L) + 0.7 × FBG (mmol/L) + 0.1 × BMI (kg/m^2^) (23).

### 2.3 Statistical analysis

All statistical analyses were conducted using R software (version 4.6.0) and Python (version 3.13.9). Continuous variables following a normal distribution were expressed as mean ± SD, whereas those not following a normal distribution were reported as median (IQR); normality was assessed using the Shapiro-Wilk test. Categorical variables were presented as counts (n) and percentages (%). Group comparisons were performed using one-way ANOVA or the Kruskal-Wallis test for continuous variables and the chi-square test or Fisher's exact test for categorical variables, as appropriate. To evaluate the association between TyHGB and AF risk, multivariable logistic regression was performed using three hierarchical models: Model 1 was unadjusted, Model 2 adjusted for age, gender, and LAD, and Model 3 additionally adjusted for smoking, hypertension, DM, CHD, LVEF, LVEDD, LVDD, and SCr. Covariates included in the multivariable models were selected based on established clinical knowledge and published literature, rather than on statistical significance in univariate analyses. This *a priori* approach was adopted to minimize the risk of both over-adjustment and residual confounding. Results are presented as odds ratios (ORs) with 95% confidence intervals (CIs). Both TyHGB and TyG were analyzed as continuous variables and as tertiles. To examine non-linear relationships, fully adjusted restricted cubic spline (RCS) regression with four knots was applied. Subgroup analyses stratified by age, gender, hypertension, DM, CHD, and LVDD were conducted, with interaction *P* values calculated. Spearman's correlation analysis was performed to assess the relationship between TyG and TyHGB. Receiver operating characteristic (ROC) curves were generated to evaluate the predictive performance of TyG, TyHGB, and their combination for AF detection, with areas under the curves (AUCs) compared using the DeLong test. The optimal cutoff value was determined using the Youden index. To assess the internal stability of our regression model and reduce the potential for overfitting, a bootstrap resampling procedure with 1,000 replicates was performed to derive bias-corrected 95% confidence intervals for the odds ratios. All tests were two-sided, and *P* < 0.05 was considered statistically significant.

## 3 Results

### 3.1 Baseline characteristics

Table 1 summarizes the baseline characteristics of the 1,137 participants included in the analysis, stratified by non-AF, PAF, and PeAF. The overall median age was 61.00 (55.00–68.00) years, with a higher proportion of males in the PeAF group (67.7%) than in the non-AF (44.3%) and PAF (54.8%) groups. Age, TC, LDL, and drinking history were comparable across the three groups, whereas all other variables differed significantly (all *P* < 0.05).

**Table 1:** Baseline characteristics.

Variables	Total (*n* = 1,137)	Non-AF (*n* = 314)	PAF (*n* = 414)	PeAF (*n* = 409)	*P*-value
Demographics					
Age, years	61.00 (55.00, 68.00)	61.00 (55.00, 68.00)	61.00 (56.00, 68.00)	62.00 (56.00, 68.00)	0.773
Men, *n* (%)	643 (56.6)	139 (44.3)	227 (54.8)	277 (67.7)	< 0.001
Smoking, *n* (%)	95 (8.4)	35 (11.1)	24 (5.8)	36 (8.8)	0.033
Drinking, *n* (%)	95 (8.4)	24 (7.6)	27 (6.5)	44 (10.8)	0.078
BMI, kg/m^2^	25.66 (23.39, 27.81)	24.49 (22.22, 27.04)	25.87 (23.68, 27.91)	26.08 (23.67, 28.20)	< 0.001
Comorbidities					
Hypertension, *n* (%)	549 (48.3)	117 (37.3)	227 (54.8)	205 (50.1)	< 0.001
DM, *n* (%)	216 (19.0)	35 (11.1)	103 (24.9)	78 (19.1)	< 0.001
CHD, *n* (%)	233 (20.5)	54 (17.2)	106 (25.6)	73 (17.8)	0.005
Stroke, *n* (%)	117 (10.3)	22 (7.0)	33 (8.0)	62 (15.2)	< 0.001
Laboratory parameters					
AST, U/L	20.70 (17.00, 25.60)	19.70 (16.30, 23.48)	20.45 (16.62, 25.50)	21.80 (18.30, 28.40)	< 0.001
ALT, U/L	19.80 (14.60, 28.60)	16.90 (12.80, 23.95)	20.20 (15.12, 30.40)	21.20 (15.60, 30.00)	< 0.001
TC, mmol/L	4.40 (3.76, 5.13)	4.46 (3.79, 5.17)	4.40 (3.78, 5.13)	4.36 (3.68, 5.04)	0.391
TG, mmol/L	1.42 (1.06, 1.97)	1.12 (0.83, 1.64)	1.67 (1.33, 2.28)	1.36 (1.03, 1.92)	< 0.001
HDL, mmol/L	1.05 (0.90, 1.21)	1.11 (0.96, 1.30)	1.02 (0.88, 1.14)	1.04 (0.90, 1.21)	< 0.001
LDL, mmol/L	2.75 (2.28, 3.31)	2.83 (2.28, 3.36)	2.73 (2.30, 3.29)	2.70 (2.23, 3.28)	0.170
SCr, umol/L	69.20 (59.50, 80.10)	63.95 (56.40, 74.28)	68.55 (59.20, 80.25)	73.00 (62.40, 82.70)	< 0.001
Uric Acid, umol/L	350.00 (295.00, 415.00)	320.00 (273.25, 365.75)	351.00 (293.25, 420.00)	377.00 (310.00, 446.00)	< 0.001
FBG, mmol/L	5.38 (4.91, 6.11)	5.19 (4.77, 5.60)	5.57 (5.04, 6.56)	5.40 (4.92, 6.23)	< 0.001
RBC, 10^12^/L	4.79 (4.42, 5.18)	4.63 (4.29, 4.95)	4.83 (4.45, 5.19)	4.95 (4.57, 5.31)	< 0.001
HGB, g/L	147.61 ± 16.54	140.77 ± 15.03	146.97 ± 15.81	153.51 ± 16.25	< 0.001
MCV, fL	90.80 (88.20, 93.70)	90.80 (88.20, 93.28)	90.00 (87.60, 92.80)	91.60 (88.80, 94.60)	< 0.001
PLT, 10^9^/L	227.00 (185.00, 266.00)	233.00 (195.00, 274.00)	223.50 (187.00, 268.00)	220.00 (179.00, 259.00)	0.005
MPV, fL	10.30 (9.70, 10.90)	10.10 (9.60, 10.70)	10.30 (9.60, 11.00)	10.40 (9.80, 10.90)	0.004
TyG	8.74 (8.45, 9.15)	8.45 (8.07, 8.86)	8.97 (8.67, 9.34)	8.70 (8.40, 9.12)	< 0.001
TyHGB	7.89 (7.11, 9.10)	7.23 (6.55, 8.07)	8.52 (7.66, 9.81)	7.86 (7.11, 9.02)	< 0.001
Echocardiographic parameters					
LAD, mm	38.00 (33.00, 42.00)	32.00 (30.00, 36.00)	36.00 (33.00, 40.00)	42.00 (40.00, 46.00)	< 0.001
LVEF, %	62.00 (58.00, 64.00)	63.00 (61.25, 65.00)	63.00 (60.00, 64.00)	59.00 (54.00, 63.00)	< 0.001
LVEDD, mm	48.00 (45.00, 51.00)	46.00 (43.00, 49.00)	48.00 (45.00, 50.00)	49.00 (46.00, 53.00)	< 0.001
LVDD, *n* (%)	608 (53.5)	64 (20.4)	173 (41.8)	371 (90.7)	< 0.001

ALT, alanine aminotransferase; AST, aspartate aminotransferase; BMI, body mass index; CHD, coronary heart disease; DM, diabetes mellitus; FBG, fasting blood glucose; HDL, high-density lipoprotein cholesterol; HGB, hemoglobin; LAD, left atrial diameter; LDL, low-density lipoprotein cholesterol; LVDD, left ventricular diastolic dysfunction; LVEDD, left ventricular end-diastolic dimension; LVEF, left ventricular ejection fraction; MCV, mean corpuscular volume; MPV, mean platelet volume; PLT, platelet count; RBC, red blood cell count; SCr, serum creatinine; TC, total cholesterol; TG, triglyceride; TyG, triglyceride-glucose index; TyHGB, triglyceride-high-density lipoprotein-glucose-body index; UricAcid, serum uric acid.

Compared with the non-AF group, the PeAF group exhibited more advanced cardiac structural remodeling, characterized by larger LAD, lower LVEF, and a stepwise increasing prevalence of LVDD (20.4% in non-AF, 41.8% in PAF, and 90.7% in PeAF, *P* < 0.001). The PAF group presented with a more adverse metabolic profile, including higher levels of TG, FBG, and TyG, as well as a higher prevalence of DM (24.9%) and CHD (25.6%), compared with the other two groups. Additionally, significant differences were observed across the three groups in height, weight, BMI, smoking history, hypertension, stroke, and most blood cell parameters, with the PeAF group showing the highest values for height, weight, BMI, and stroke prevalence. For subsequent analyses, PAF and PeAF were combined into a single AF group.

### 3.2 Multivariable logistic regression analysis

Multivariable logistic regression was performed using three hierarchical models (Table 2). Model 1 was unadjusted. Model 2 adjusted for age, gender, and LAD. Model 3 further adjusted for smoking, hypertension, DM, CHD, LVEF, LVEDD, LVDD, and SCr. Both TyHGB and TyG were significantly associated with AF risk across all models (all *P* < 0.001). In the fully adjusted model, each one-unit increase in TyHGB was associated with a 61% increase in the odds of AF (OR 1.61, 95% CI 1.41–1.85). When analyzed by tertiles, a dose-response relationship was evident for both indices. In Model 3, compared with the lowest tertile, the ORs for the highest tertile of TyHGB and TyG were 4.28 (95% CI 2.74–6.71) and 7.09 (95% CI 4.54–11.07), respectively. Together, these findings indicate that both TyHGB and TyG are independently associated with AF.

**Table 2:** Multivariable logistic regression analysis of TyHGB and TyG with AF risk.

Categories	Model 1		Model 2		Model 3	
	OR (95% CI)	*P*-value	OR (95% CI)	*P*-value	OR (95% CI)	*P*-value
**TyHGB**	1.70 (1.51–1.90)	< 0.001	1.60 (1.41–1.82)	< 0.001	1.61 (1.41–1.85)	< 0.001
T1 ( ≤ 7.37)	Reference		Reference		Reference	
T2 (7.37–8.58)	2.28 (1.68–3.10)	< 0.001	2.24 (1.56–3.23)	< 0.001	2.21 (1.52–3.22)	< 0.001
T3 (8.58)	5.49 (3.81–7.90)	< 0.001	4.49 (2.97–6.79)	< 0.001	4.28 (2.74–6.71)	< 0.001
**TyG**	4.95 (3.67–6.68)	< 0.001	6.79 (4.69–9.84)	< 0.001	7.22 (4.89–10.65)	< 0.001
T1 ( ≤ 8.55)	Reference		Reference		Reference	
T2 (8.55–9.00)	3.35 (2.43–4.60)	< 0.001	5.27 (3.54–7.85)	< 0.001	5.75 (3.80–8.72)	< 0.001
T3 (>9.00)	5.27 (3.72–7.48)	< 0.001	6.78 (4.44–10.36)	< 0.001	7.09 (4.54–11.07)	< 0.001

Model 1: Unadjusted. Model 2: Adjusted for age, gender, and left atrial diameter. Model 3: Adjusted for age, gender, left atrial diameter, smoking, hypertension, diabetes mellitus, coronary heart disease, left ventricular ejection, left ventricular end-diastolic dimension, left ventricular diastolic dysfunction, and serum creatinine. CI, confidence interval; OR, odds ratio; TyG, triglyceride-glucose index; TyHGB, triglyceride-high-density lipoprotein-glucose-body index.

### 3.3 RCS analysis

RCS analysis adjusted for covariates in Model 3 revealed significant non-linear associations of both TyHGB and TyG with AF risk (*P* for non-linear < 0.001 for both; Figures 2A, D). The same non-linear patterns held in the PAF subgroup (Figures 2B, E). Corresponding positive non-linear associations between TyHGB, TyG and PeAF risk were also observed (Figures 2C, F).

**Figure 2:**
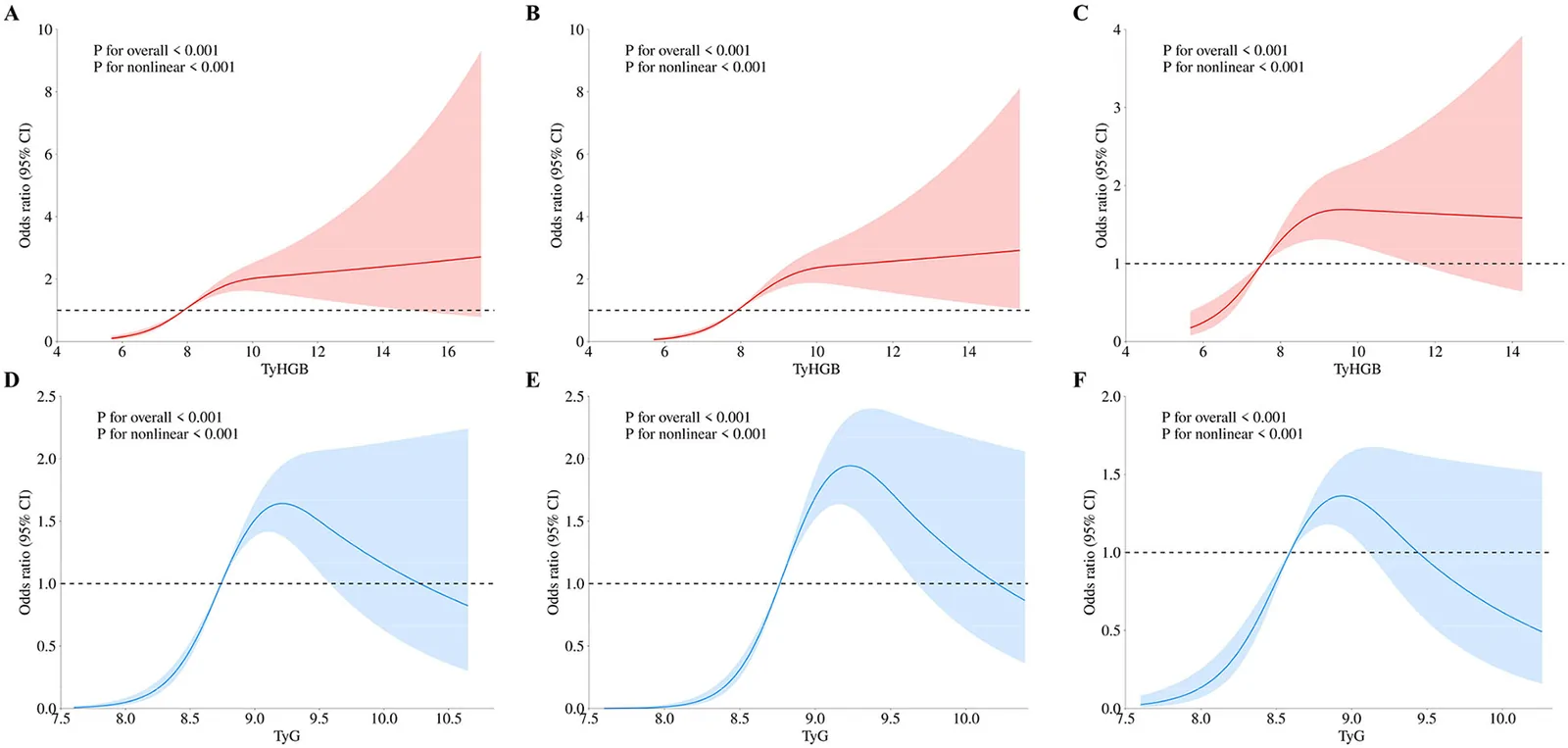
Association between TyHGB and TyG with AF and its subgroups (Adjusted Model 3). **(A-C)** RCS curves for the association between TyHGB and risks of overall AF, PAF and PeAF; **(D-F)** RCS curves for the association between TyG and risks of overall AF, PAF and PeAF.

### 3.4 Subgroup analysis

Subgroup analyses were performed to assess the robustness of the associations between TyHGB and AF risk across predefined clinical strata. When TyHGB was analyzed as a continuous variable (Figure 3A), each 1-unit increment in TyHGB was significantly linked to elevated AF odds across all predefined subgroups (all *P* < 0.001). The overall adjusted OR was 1.70 (95% CI: 1.51–1.90). We further categorized TyHGB into tertiles to assess dose-response relationships. Relative to the lowest tertile (T1), the middle tertile (T2) conferred an OR of 2.28 (95% CI: 1.68–3.10, Figure 3B), and the highest tertile (T3) yielded an OR of 5.49 (95% CI: 3.81–7.90, Figure 3C). Consistently significant positive associations were observed within every stratum for both T2 vs. T1 and T3 vs. T1 comparisons (all *P* < 0.05 for individual subgroups). All interaction *P* were > 0.05 across the three analyses, suggesting the association between TyHGB and AF was not modified by the examined clinical factors. These findings confirm the stability and generalizability of the independent association between TyHGB and AF.

**Figure 3:**
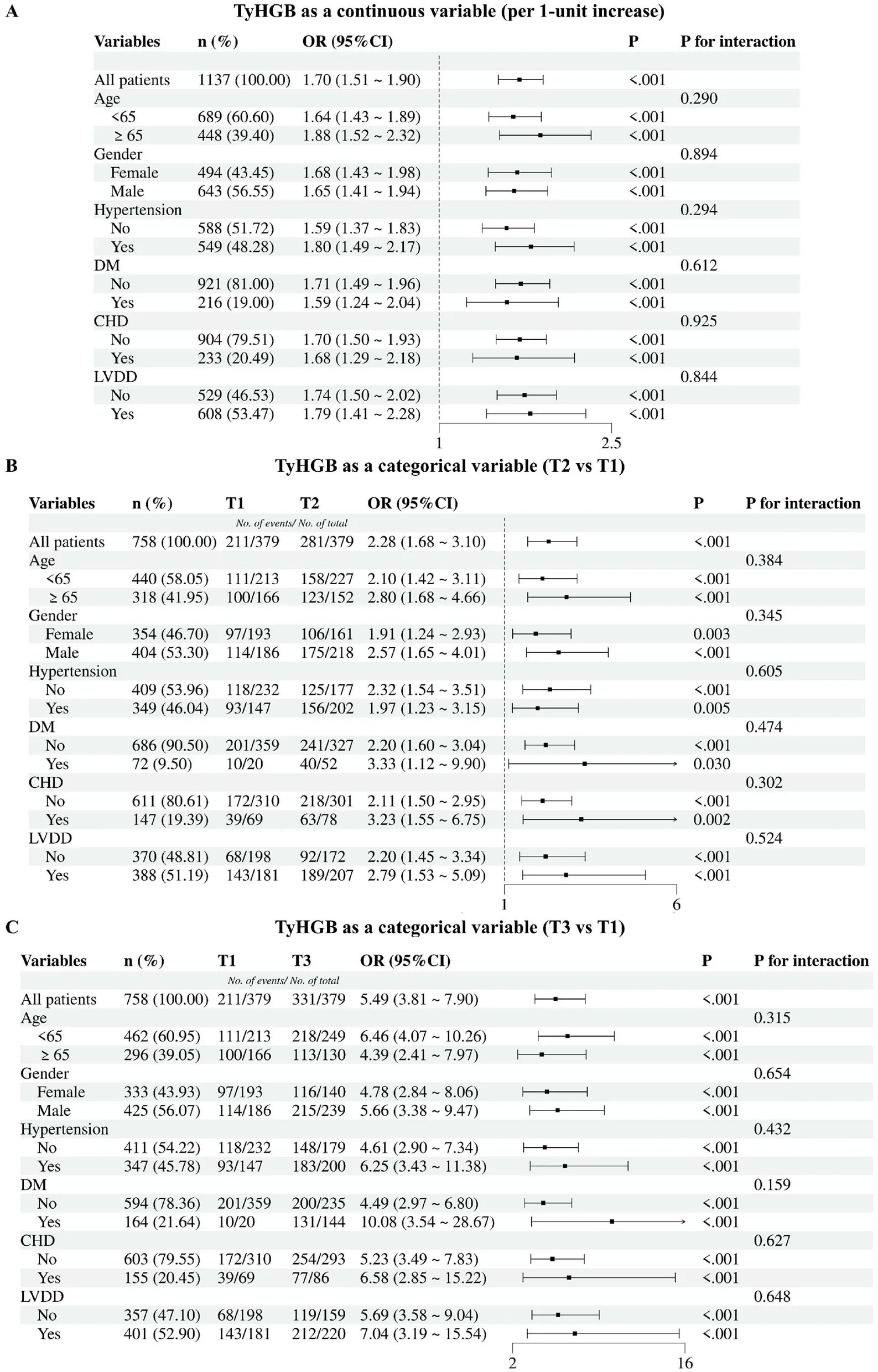
Subgroup analyses of the association between TyHGB and AF risk. **(A)** TyHGB as a continuous variable (per 1-unit increase); **(B)** TyHGB as a categorical variable (T2 vs. T1); **(C)** TyHGB as a categorical variable (T3 vs. T1).

### 3.5 ROC curve analysis

ROC curve analyses revealed moderate predictive performance of TyG and TyHGB for AF, with AUC values of 0.717 (95% CI: 0.680–0.752) and 0.716 (95% CI: 0.683–0.750), respectively (Figure 4). DeLong test verified no significant difference in predictive efficacy between the two single indicators (*P* = 0.902). As shown in Table 3, the combined model of TyG and TyHGB achieved the highest AUC (0.723) and Youden index (0.363). Further comparison demonstrated that the combined model was significantly superior to TyG alone (*P* = 0.002), while its predictive ability was statistically comparable to TyHGB (*P* = 0.366). These data suggest that combining TyG with TyHGB can effectively optimize the predictive value for AF.

**Figure 4:**
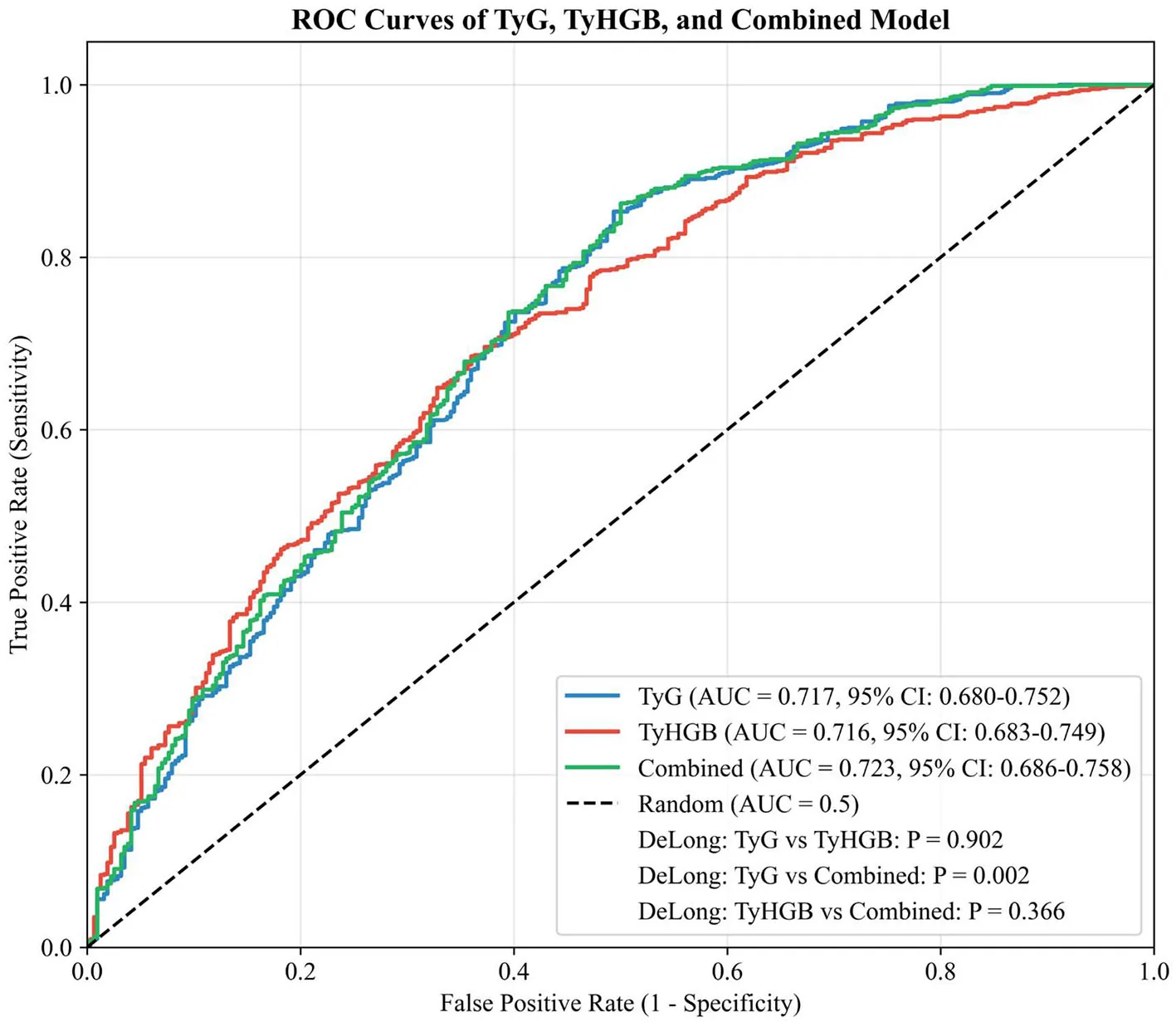
ROC curves of TyG, TyHGB, and their combination for predicting AF risk.

**Table 3:** Comparison of the ability of TyHGB, TyG, and their combination to predict AF risk.

Model	AUC	95% CI	Sensitivity	Specificity	Youden index	Cutoff
TyHGB	0.716	0.683–0.749	0.685	0.640	0.325	7.563
TyG	0.717	0.680–0.752	0.853	0.506	0.359	8.448
TyHGB + TyG	0.723	0.686–0.758	0.863	0.500	0.363	0.626

AUC, area under the curve; CI, confidence interval; TyG, triglyceride-glucose index; TyHGB, triglyceride-high-density lipoprotein-glucose-body index.

### 3.6 Correlation analysis

We further analyzed the correlation between TyG and TyHGB (Figure 5). Spearman correlation analysis revealed a significant strong positive correlation (ρ = 0.870, *P* < 0.001), indicating a positive monotonic association between the two indicators.

**Figure 5:**
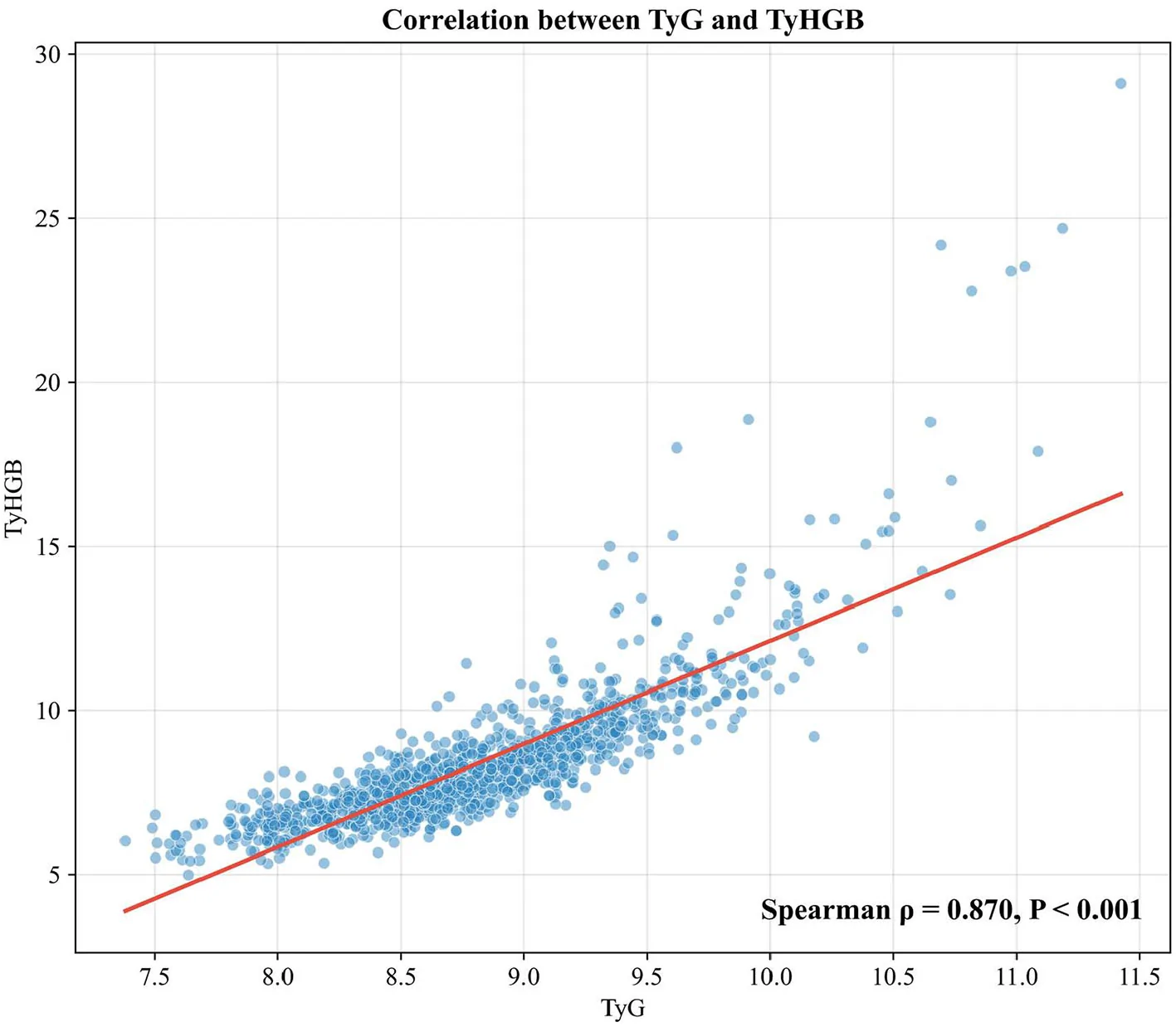
Correlation analysis between TyHGB and TyG.

### 3.7 Bootstrap internal validation

To assess the internal stability of our regression model, a bootstrap resampling procedure with 1,000 replicates was performed. The median OR from 1,000 resamples was 1.63 (95% CI: 1.34–2.01), which was consistent with the primary analysis (OR = 1.61, 95% CI: 1.41–1.85), supporting the internal stability of our estimates.

## 4 Discussion

In this retrospective case-control study, we evaluated the association between the novel metabolic index TyHGB and AF risk. Three main findings emerged. First, TyHGB was independently associated with AF, exhibiting a significant non-linear dose-response relationship. Second, its predictive performance was comparable to the well-established TyG index. Third, the combination of TyG and TyHGB further improved AF detection compared with TyG alone, although it did not outperform TyHGB alone.

To our knowledge, this is the first study to examine the association between TyHGB and AF. In our study population, TyHGB demonstrated a predictive accuracy comparable to TyG (AUC 0.716 vs. 0.717, *P* = 0.902). This suggests that TyG, as a core indicator of insulin resistance, already captures a substantial portion of the metabolic risk for AF. The additional inclusion of BMI did not significantly enhance discriminative ability, possibly because insulin resistance is the predominant driver of AF risk in our population. The combined model of TyG and TyHGB achieved the highest AUC (0.723) and significantly outperformed TyG alone (*P* = 0.002), though it was not superior to TyHGB alone (*P* = 0.366). Given the strong positive correlation between TyG and TyHGB (ρ = 0.870, *P* < 0.001), indicating substantial overlap in the metabolic information captured by the two indices, the incremental predictive value of the combined model over TyHGB alone is limited. From a clinical standpoint, we do not advocate for the routine combined use of both indices; rather, TyHGB alone may serve as a practical and integrated metabolic marker for risk assessment. Future studies comparing TyHGB with a broader range of insulin resistance-related indices, such as the homeostatic model assessment of insulin resistance (HOMA-IR) or the metabolic score for insulin resistance (METS-IR), are warranted to further delineate its relative performance.

Insulin resistance, reflected by the TG/HDL ratio and FBG, is a central feature of metabolic syndrome and has been linked to AF risk in previous studies (24, 25). Chronic insulin resistance promotes a pro-inflammatory state characterized by elevated levels of tumor necrosis factor-alpha, interleukin-6, and C-reactive protein (26). These inflammatory cytokines can affect atrial myocytes, promoting electrical instability and facilitating re-entry circuits, thereby creating a substrate for AF initiation and maintenance (26). Insulin resistance also induces oxidative stress through the overproduction of reactive oxygen species from mitochondria and NADPH oxidases (27). Excessive reactive oxygen species can impair intracellular calcium handling by affecting ryanodine receptors and sarcoendoplasmic reticulum calcium ATPase, alter ion channel function, and promote atrial fibrosis through activation of transforming growth factor-beta signaling pathways (28). Autonomic dysfunction, another consequence of insulin resistance, can increase sympathetic tone and reduce heart rate variability, both of which have been associated with increased AF susceptibility (29, 30). These interconnected pathways provide biological plausibility for the association between the metabolic components of TyHGB and AF risk (7).

Obesity, captured by the BMI component of TyHGB, contributes to atrial remodeling through several distinct but interconnected pathways (31). EAT, which is markedly increased in obese individuals, has gained recognition as an important mediator of obesity-related AF risk (32). Unlike subcutaneous fat, EAT shares the same microcirculation as the underlying atrial myocardium, with no fascial barrier separating them (33). This unique anatomical proximity allows EAT to directly influence atrial electrophysiology and structure. EAT is not merely a passive fat depot but an active endocrine organ that secretes a wide range of pro-inflammatory adipokines, including leptin, resistin, and plasminogen activator inhibitor-1, as well as free fatty acids and reactive oxygen species (34, 35). These secreted factors can infiltrate the adjacent atrial wall, promoting local inflammation, fibroblast proliferation, collagen deposition, and conduction heterogeneity (36, 37). Clinical imaging studies using cardiac magnetic resonance and computed tomography have suggested that increased EAT volume is associated with higher AF incidence and greater recurrence after catheter ablation (38). Beyond EAT-mediated mechanisms, obesity also increases hemodynamic burden, leading to atrial enlargement, elevated left atrial filling pressures, and increased wall stretch (39). These mechanical changes activate stretch-activated ion channels, promote fibroblast proliferation, and upregulate extracellular matrix remodeling, all of which may contribute to a pro-arrhythmogenic substrate (40).

An important advantage of TyHGB is that it captures the synergistic effects of concurrent metabolic abnormalities that frequently coexist in the same individual. In clinical practice, insulin resistance, dyslipidemia, hyperglycemia, and obesity rarely occur in isolation; rather, they often coexist as components of the metabolic syndrome (41). Compared with individuals with isolated insulin resistance or isolated obesity, those with both conditions simultaneously have higher AF risk (42). The combination of hyperglycemia, dyslipidemia, and excess adiposity may have additive or synergistic effects on inflammatory pathways and oxidative stress (43). For example, hyperglycemia exacerbates obesity-induced inflammation by promoting the formation of advanced glycation end-products, which bind to their receptors and activate nuclear factor-kappa B (NF-κB) signaling (44). Conversely, obesity can worsen insulin resistance through adipose-derived free fatty acids and adipokines, creating a cycle that amplifies metabolic dysregulation. By integrating insulin resistance, glucose metabolism, and adiposity into a single composite index, TyHGB reflects this cumulative, synergistic metabolic burden across multiple dimensions (45). This integrative property is particularly relevant given that AF is a multifactorial disease resulting from the interplay of multiple metabolic abnormalities rather than a single isolated risk factor.

From a clinical perspective, TyHGB is simple, low-cost, and readily available, as it can be calculated from routine laboratory tests (TG, HDL, FBG) and basic physical examination (BMI), without requiring specialized equipment or additional patient visits, making it a convenient complement to TyG. Although TyHGB has practical advantages, it should not be used alone for AF screening given its modest AUC. Instead, it may contribute to a more comprehensive metabolic risk assessment when combined with other clinical risk factors, particularly in individuals with obesity or suspected metabolic syndrome.

Several limitations should be acknowledged. First, the retrospective design precludes causal inference. Specifically, the higher prevalence of hypertension, DM, and CHD in the PAF group compared with the PeAF group may have introduced some bias when evaluating the association between TyHGB and AF subtypes. Second, the single-center nature and modest sample size may limit the generalizability of our findings. Future studies with larger, multicenter cohorts are warranted. Third, our control group was restricted to patients with PSVT, selected to achieve comparable symptom profiles and diagnostic workup between groups. Although this approach improves internal validity by minimizing symptom- and workup-related confounding, it limits the generalizability of our results to other hospitalized non-AF populations. Further studies employing more diverse control cohorts are needed to validate and extend our observations. Fourth, TyHGB was based on single measurements at hospital admission, without exploring temporal changes or the impact of metabolic interventions on clinical outcomes. Fifth, detailed information on medication use, including glucose-lowering agents, lipid-lowering therapies, anticoagulants, and rate or rhythm control medications, was not available in our retrospective database. These medications could potentially influence both metabolic parameters and AF risk, representing a source of unmeasured confounding that cannot be excluded. Sixth, our classification of AF subtypes was based solely on the duration of episodes according to current guidelines. We did not perform sub-analyses stratified by ventricular rate, such as rapid ventricular response, which could be a clinically relevant factor. Future studies with larger sample sizes designed to investigate the interaction between heart rate control and metabolic indices in AF risk are warranted. Seventh, TyG and TyHGB may be influenced by a variety of factors beyond metabolic status, including dietary intake, nutritional status, inflammation, and psychological factors. Data on dietary patterns, depressive symptoms, frailty, and related medications were not systematically recorded in our retrospective database. While we acknowledge that these factors were not adjusted for in our analysis, we elected not to incorporate them for two reasons: first, these factors are interconnected with metabolic status through complex pathways, and adjusting for them could lead to over-adjustment; second, our study was designed to evaluate TyHGB as a simple and clinically accessible screening tool, rather than to establish a comprehensive etiological model. We recognize this as a clear limitation, and future prospective studies with comprehensive lifestyle, nutritional, and psychological assessments are warranted to confirm the robustness of our findings. Eighth, although we restricted enrollment to clinically stable patients and collected blood samples under standardized fasting conditions, we cannot entirely exclude the possibility that transient metabolic disturbances related to hospitalization may have influenced TyG and TyHGB levels. This represents a potential source of measurement bias inherent to retrospective hospital-based studies. Additionally, the bootstrap analysis was performed primarily as an internal validation to assess the stability of our model estimates; external validation in prospective, multi-center cohorts is still needed to confirm the generalizability of our findings.

## 5 Conclusion

TyHGB is a novel metabolic index that integrates insulin resistance, glucose metabolism, and adiposity. It is independently associated with AF risk and demonstrates predictive performance comparable to the established TyG index. As a convenient, low-cost complementary marker providing additional information on adiposity, TyHGB may facilitate more comprehensive metabolic risk assessment in clinical practice. Future studies should further evaluate the clinical utility of TyHGB and integrate it with other cardiovascular biomarkers to develop more comprehensive risk stratification strategies for AF.

## Data Availability

The original contributions presented in the study are included in the article/Supplementary material, further inquiries can be directed to the corresponding authors.
